# Mitochondrial CDP-diacylglycerol synthase activity is due to the peripheral protein, TAMM41 and not due to the integral membrane protein, CDP-diacylglycerol synthase 1

**DOI:** 10.1016/j.bbalip.2017.12.005

**Published:** 2018-03

**Authors:** Nicholas J. Blunsom, Evelyn Gomez-Espinosa, Tim G. Ashlin, Shamshad Cockcroft

**Affiliations:** Dept. of Neuroscience, Physiology and Pharmacology, Division of Biosciences, University College London, London WC1E 6JJ, UK

**Keywords:** CDS, CDP diacylglycerol synthase, PA, phosphatidic acid, PI, phosphatidylinositol, PC, phosphatidylcholine, PGC-1α, peroxisome proliferator-activated receptor γ coactivator 1α, CDP-DG, CDP-diacylglycerol, PITPNC1, phosphatidylinositol transfer protein cytosolic 1, RA, retinoic acid, DG, diacylglycerol, FCS, fetal calf serum, cyto *c*, cytochrome C, FCCP, carbonyl cyanide 4-(trifluoromethoxy)phenylhydrazone, mETS, maximal electron transport system, nmt, non-mitochondrial, ER, endoplasmic reticulum, MAMs, mitochondrial-associated endoplasmic reticulum membranes, WCL, whole cell lysates, micro, microsomes, PIS, PI synthase, COXIV, cytochrome *c* oxidase subunit IV, GRP75, 75 kDa Glucose-regulated protein, Mitochondria, Differentiation, Retinoic acid, Phosphatidic acid, Cardiolipin, Heart, PITPNC1

## Abstract

CDP diacylglycerol synthase (CDS) catalyses the conversion of phosphatidic acid (PA) to CDP-diacylglycerol, an essential intermediate in the synthesis of phosphatidylglycerol, cardiolipin and phosphatidylinositol (PI). CDS activity has been identified in mitochondria and endoplasmic reticulum of mammalian cells apparently encoded by two highly-related genes, *CDS1* and *CDS2*. Cardiolipin is exclusively synthesised in mitochondria and recent studies in cardiomyocytes suggest that the peroxisome proliferator-activated receptor γ coactivator 1 (PGC-1α and β) serve as transcriptional regulators of mitochondrial biogenesis and up-regulate the transcription of the *CDS1* gene. Here we have examined whether CDS1 is responsible for the mitochondrial CDS activity. We report that differentiation of H9c2 cells with retinoic acid towards cardiomyocytes is accompanied by increased expression of mitochondrial proteins, oxygen consumption, and expression of the PA/PI binding protein, PITPNC1, and CDS1 immunoreactivity. Both CDS1 immunoreactivity and CDS activity were found in mitochondria of H9c2 cells as well as in rat heart, liver and brain mitochondria. However, the CDS1 immunoreactivity was traced to a peripheral p55 cross-reactive mitochondrial protein and the mitochondrial CDS activity was due to a peripheral mitochondrial protein, TAMM41, not an integral membrane protein as expected for CDS1. TAMM41 is the mammalian equivalent of the recently identified yeast protein, Tam41. Knockdown of TAMM41 resulted in decreased mitochondrial CDS activity, decreased cardiolipin levels and a decrease in oxygen consumption. We conclude that the CDS activity present in mitochondria is mainly due to TAMM41, which is required for normal mitochondrial function.

## Graphical Abstract

**Figure:**
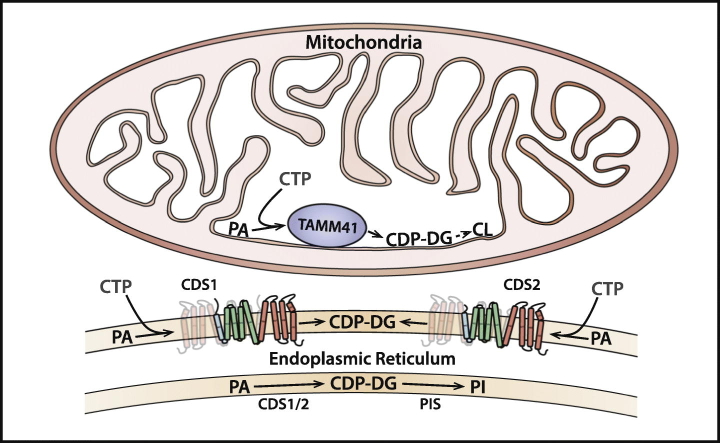

## 1 Introduction

The heart has the highest levels of cardiolipin; it comprises as much as 15–20% of the entire phospholipid phosphorus mass of that organ [1]. This reflects the high mitochondrial density, and in cardiomyocytes there are several thousand mitochondria that are arranged in a regular pattern. Mitochondria are located around the nucleus, between the myofibrils and beneath the sarcolemma, and occupy nearly a third of the volume of a cardiomyocyte [2], [3]. The transcriptional regulators, PGC-1α and β have been shown to serve as master regulators of mitochondrial biogenesis and function [4], [5] and in mice, combined deficiency of both revealed cardiolipin deficiency [6]. This phenotype was correlated with PGC-1α-mediated transcriptional control of CDP-diacylglycerol synthase 1 (CDS1), an enzyme that catalyses the conversion of PA to CDP-DG, an essential intermediate step in the biosynthesis of cardiolipin and phosphatidylinositol. Gene expression profiling of PGC-1α/β^−/−^ hearts revealed reduced expression of CDS1. Moreover, *CDS1* gene promoter-reporter co-transfection experiments and chromatin precipitation studies demonstrated that PGC-1α co-regulates estrogen-related receptors to activate the transcription of the *CDS1* gene [6]. In mammals, two homologous genes of *CDS* (*CDS1* and *CDS2*) have been cloned that are 73% identical and 92% similar [7], [8], [9]. Human CDS1 is 461 amino acids long and has a calculated molecular weight of ~ 53 kDa whilst CDS2 is 444 amino acids with a calculated molecular weight of ~ 51 kDa. *CDS* genes homologous to mammalian Cds are found in *E. coli*, *S. cerevisiae*, *Drosophila*, zebrafish, plants and cyanobacteria [9], [10], [11], [12], [13], [14], [15], [16]. The structure of Cds from the bacterium, *Thermotoga maritama* (TmCdsA) indicates a homodimer and each monomer contains nine transmembrane helices arranged into a novel fold with three domains [17] (see Fig. 5B). An unusual funnel-shaped cavity penetrates half way into the membrane, allowing the enzyme to simultaneously accept CTP and PA.

In mammalian cells, over-expressed CDS1 and CDS2 localise at the endoplasmic reticulum (ER) [7], [18], [19]. Yet, previous studies in rat liver and lung have indicated the presence of mitochondrial as well as microsomal CDS activity [20], [21], [22], [23]. Moreover, expression studies indicate that CDS1 and CDS2 exhibit quite different tissue specificity [7]. In the mouse, CDS2 appears to be ubiquitously expressed whilst CDS1 has a restricted pattern of expression. CDS1 has been shown to be highly expressed in the heart [24] and in SHHF (spontaneously hypertensive heart failure) rats, an increase in CDS1 mRNA was observed with increasing age whilst CDS2 mRNA decreased during heart failure development [25]. The increase in CDS1 mRNA corresponded to an increase in mitochondrial CDS activity with no change in microsomal CDS activity [25].

In yeast, a single Cds protein, Cds1, has been characterised which is localised exclusively at the ER [26], [27]. A recent study in *S. cerevisiae* has identified that Tam41 (Translocator maintenance protein), a mitochondrial inner membrane peripheral protein functions as a CDP-DG synthase for the synthesis of cardiolipin [26]. TAM41 was first identified for its role in maintaining the integrity and activity of the protein import complexes, TIM23 and TIM22 [28]. However, in the absence of Tam41, some cardiolipin was produced indicating that yeast Cds1 could supply CDP-DG for cardiolipin synthesis. The aim of the present work was to identify whether CDS1 was responsible for the CDS activity found in mitochondria of mammalian cells including the heart. Our recent studies have identified a lipid transporter, PITPNC1, to be enriched in the heart [29], [30]. PITPNC1 is a PA/PI binding protein [31] and could therefore supply PA for CDP-DG synthesis. In this study, we have used an in vitro H9c2 cell model along with mitochondria from rat heart, liver and brain to demonstrate that the mitochondrial CDS activity is due to a peripheral protein, TAMM41, a mammalian homologue of the recently-identified yeast Tam41 [26].

## 2 Material and methods

### 2.1 Materials

The following antibodies were purchased from Santa Cruz Biotechnology: cytochrome *c* (6H2): sc-13561 mouse monoclonal, Troponin I (H-170): sc-15368 rabbit polyclonal and anti-Myc antibody 9E10: sc-40 mouse monoclonal. The CDS1 antibody (CDP-diacylglycerol synthase 1, mouse monoclonal) was purchased from either Abcam (ab88121) or Novus Biologicals (Clone 2D10) as the same antibody is sold by both companies. COXIV, rabbit polyclonal (#4844) was purchased from Cell Signalling Technology; GRP75 (mortalin) Clone N52A/42 mouse monoclonal was obtained from BioLegend; Calnexin rabbit polyclonal was obtained from Enzo Life Sciences (ADI-SPA-865). The antibody to PITPNC1 (RdgBβ), affinity-purified rabbit polyclonal antibody (Rb59) was a gift from Nils Halberg (Tavazoie lab), Rockefeller University [32]. The peptide used to generate the antibody was DPEKKATLNLPGMHSSDK (308–325a.a.) and therefore only recognises the long form, splice variant 1 [30]. The antibody was characterised further; it recognised recombinant protein and over-expressed protein and finally knockdown of PITPNC1 by siRNA led to loss of the appropriate band on the gel. Antibodies to PITPα (PAb:674) and PITPβ (MAb:4A7) were made in-house and PAb:674 has been described previously [33]. The TAMM41 antibody (HPA036834) was purchased from ATLAS antibodies. In Table 1, we provide a summary of the antibodies used in this paper and whether the antibody has been validated for western blotting. In many cases there are significant number of publications where the antibody has been used as a marker for an organelle or as a loading control but no formal validation has been done to our knowledge. siRNA for rat CDS1_1 (Cat. No. SI01497811) rat CDS1_3 (Cat. No. SI01497825); rat CDS2_1 (Cat. No. SI01497839) and rat CDS2_3 (Cat. No. SI01497853) and rat TAMM41_1 (Cat. No. SI02905798) and rat TAMM41_2 (Cat. No. SI02905805), negative control siRNA (Cat No. 1027310) were obtained from Qiagen.

**Table 1:** Summary of antibodies used in this paper. All antibodies used here were either commercially obtained or made in-house. Only some of the antibodies have been formally validated for western blotting. However, several of the antibodies have been cited in many publications.

Antibodies	Company	Code	Species	Comments and references
Cytochrome c	Santa Cruz Biotechnology	sc-13561 (6H2)	Mouse monoclonal	Antibody used in 59 publications (see https://www.scbt.com/scbt/product/cytochrome-c-antibody-6h2?requestFrom=search) but not validated by knockdown and by overexpression.
Troponin I	Santa Cruz Biotechnology	sc-15368 (H-170)	Rabbit polyclonal	Antibody used in 19 publications (see https://www.scbt.com/scbt/product/troponin-i-antibody-h-170?requestFrom=search) but not validated by knockdown and by overexpression.
Myc	Santa Cruz Biotechnology	sc-40 (9E10)	Mouse monoclonal	Antibody used in > 2900 publications (see https://www.citeab.com/antibodies/2390803-sc-40-c-myc-antibody-9e10) and validated by over-expression.
COXIV	Cell Signalling Technology	#4844	Rabbit polyclonal	Antibody used in 110 publications as a marker for mitochondria or as a loading control (see https://www.citeab.com/antibodies/124783-4844-cox-iv-antibody) but not validated by knockdown and by overexpression.
GRP75 (mortalin)	BioLegend	Clone N52A/42	Mouse monoclonal	See http://neuromab.ucdavis.edu/datasheet/N52A_42.pdf for validation by siRNA
Calnexin	Enzo Life Sciences	ADI-SPA-865	Rabbit polyclonal	Antibody used in 29 papers as a marker for ER or as a loading control (see https://www.citeab.com/antibodies/307955-adi-spa-865-calnexin and http://www.enzolifesciences.com/ADI-SPA-865/calnexin-polyclonal-antibody/) but not validated by knockdown and by overexpression.
CDS1	Abcam	Ab88121	Mouse monoclonal (clone 2D10)	Recognises over-expressed protein only; in animal tissues only recognises an unknown peripheral mitochondrial protein p55 (this paper). Antibody used previously in two papers very likely recognises the p55 mitochondrial protein [43], [44].
	Novus Biologicals	H00001040-M01		
TAMM41	ATLAS antibodies	HPA036834	Rabbit polyclonal	This paper, siRNA knockdown of band of appropriate size.
PITPNC1 (RdgBβ)	Gift from Tavozie Lab	Pab:Rb59	Rabbit polyclonal (purified)	siRNA knockdown and recognition of recombinant and over-expressed protein (performed in Cockcroft Lab) [32].
PITPα	Cockcroft Lab	Pab:674	Rabbit polyclonal (serum)	siRNA knockdown and recognition of recombinant and over-expressed protein (performed in Cockcroft Lab).
PITPβ	Cockcroft Lab	Mab:4A7	Mouse monoclonal	siRNA knockdown and recognition of recombinant and over-expressed protein (performed in Cockcroft Lab).

### 2.2 H9c2 cell culture and differentiation and transfection of H9c2 cells with siRNA

H9c2 cells (ATCC number CRL-1446) derived from embryonic BD1X rat ventricular tissue were purchased from ATCC. The cells were cultured in Dulbecco's modified Eagle's medium (DMEM) 1X w/GlutaMAX + Glucose + NaPyr. (Cat. No. 31966-021) supplemented with 10% heat inactivated fetal calf serum (FCS) and 0.5 iu·mL^− 1^ penicillin and 50 μg·mL^− 1^ streptomycin at 37 °C with 10% CO_2_. Before 70% confluence, cells were split and seeded at 1.1 × 10^6^ cells in T175 flask (175 cm^2^).

For differentiation, 5 × 10^5^ cells were plated in 10 cm dishes with 10% FCS culture medium and cultured for 2 days. Once 80% confluency was reached, cells were cultured in medium with 1% FCS and daily addition of 1 μM all-*trans*-retinoic Acid (Sigma). The culture medium was replaced every 2 days for a total of 8–12 days.

Proliferating H9c2 cells were seeded in 2 × T175 flasks (10^6^ cells per flask) and transfected with a mixture of two siRNA (10 nM total) with HiPerFect Transfection Reagent following the manufacturer instructions (Qiagen). Cells were incubated for 72 h to achieve confluency (~ 8 × 10^6^ cells) prior to be used for experiments [34].

### 2.3 Immunofluorescence protocol

H9c2 cells were grown on glass coverslips (19 mm, thickness 0) for 72 h. Cells were washed in phosphate-buffered saline (PBS) and subsequently fixed with 4% paraformaldehyde in PBS, pH 7.4 with 100 mM MgCl_2_ and 100 mM CaCl_2_ for 15 min at room temperature. Following a further two washes in PBS, cells were permeabilised with PBS containing 0.2% Triton X-100 and 100 mM glycine for 10 min at room temperature. Cells were then washed again three times, before blocking with 0.1% BSA in PBS with 100 mM glycine, for 30 min at room temperature. In order to stain for nuclei and actin, 49,6-diamidino-2-phenylindole (DAPI) (400 μg/ml) and Rhodamine-phalloidin (2 nM) was added for 30 min at room temperature in the dark. Following incubation with the chemical stains, cells were washed three times with blocking solution, three times with PBS, and finally rinsed with d.H_2_O before mounting on a microscope slide with Fluoroshield (Sigma, F6182).

### 2.4 COS-7 cells culture and transfection using FuGene HD

The plasmids pcDNA3.1-Myc-CDS1 and pcDNA3.1-Myc-CDS2 was provided by Dr Tamas Balla (NICHD Bethesda, MD, USA) [18]. COS-7 cells were maintained in DMEM supplemented with 10% FCS and 0.5 iu·mL^− 1^ penicillin and 50 μg·mL^− 1^ streptomycin at 37 °C with 5% CO_2_. COS-7 cells were transfected with FuGENE HD (Promega) as per manufacturer's protocol. The cell media was changed 24 h after transfection. Cells were generally incubated for 72 h prior to use. For western blot analysis of CDS, COS-7 cell membranes were used. The COS-7 cells were sonicated and the lysate was centrifuged at 100,000*g* for 1 h. at 4 °C to pellet total membranes.

### 2.5 Western blotting

H9c2 cell were harvested in RIPA buffer supplemented with protease inhibitors (Sigma, P8340). The protein concentration of the lysates was determined using the BCA (bicinchoninic acid) assay and the proteins (50 μg) were separated by SDS PAGE on 12% acrylamide gels made in-house. When analysing myc-tagged CDS proteins, Invitrogen NuPAGE 4–12% Bis-Tris gels had to be used because the myc-tagged proteins failed to enter the resolving gel in the gels prepared in-house. After western blotting was completed, Ponceau S staining of the membrane was carried out as a loading control. (The profile of staining with Ponceau S before or after performing the western blot is near identical). The membrane was incubated with Ponceau S solution (Sigma) for 5 min. The membrane was then washed and imaged under blue light. For western blot, antibodies were used at the following dilutions: CDS1 (1:100); Troponin I (1: 200), PITPNC1 (1:100), COXIV (1:1000), cytochrome *c* (1:1000), GRP75 (1:1000), calnexin (1:1000), PITPα (1:1000), PITPβ (1:1000) and TAMM41 (1:100).

### 2.6 Measurement of oxygen consumption

Mitochondrial oxygen consumption was analysed using an Oroboros Oxygraph-2 k as described [35]. Cells were detached using trypsin (Gibco), harvested and resuspended in HEPES buffer (137 mM NaCl, 3.7 mM KCl, 2 mM MgCl_2_, 1 mM CaCl_2_, 1 mg/ml BSA and 5.6 mM Glucose, pH 7.4). Experimental temperature was maintained at 37 °C, and oxygen concentration readings from the medium were calibrated according to manufacturer's protocol. 2 ml of H9c2 cells (1–1.6 × 10^6^ cells per ml) were added to the chamber. After reading basal oxygen consumption measurements (Routine Respiration), oligomycin 2.5 μM (Sigma) was added to measure Leak respiration. Initial addition of 2 μM followed by titrations of 0.5 μM of the uncoupler Carbonyl cyanide 4-(trifluoromethoxy)phenylhydrazone (FCCP) (Sigma) was used to induce and record maximal uncoupled respiration (mETS, electron transport system, uncoupled). Finally, addition of 2.5 μM antimycin A (Sigma) allowed the measurement of any non-mitochondrial residual oxygen consumption (nmt).

### 2.7 RNA isolation and real time PCR assay

Total RNA was prepared using the Spin Column Total RNA Miniprep Kit from Qiagen following manufacturer instructions. RNA (1 μg) was reverse transcribed into cDNA using SuperScript™ II Reverse Transcriptase and random hexamer primers (Invitrogen). Real-time quantitative PCR analysis was performed using KAPA SYBR® FAST qPCR kit Master Mix (KAPABIOSYSTEMS) and primers. (Specific *Rattus norvegicus* primers were designed using the website Primer 3 based on the NCBI sequences - available on request). Quantitative PCR was performed using the CFX96 instrument (BioRad) and transcript levels were determined using the 2^− ΔΔCt^ method and normalized to PGK1 transcript levels [36]. All PCR reactions were performed in triplicate.

### 2.8 CDS and PI synthase activity

CDP diacylglycerol synthase (CDS) activity on isolated tissue and cellular samples was determined by measuring the level of incorporation of [^3^H]-CTP into CDP-DG using external phosphatidic acid as described previously [19]. CDS activity was measured in a 100 μl assay containing 50 mM Tris-HCl (pH 8), 74 mM KCl, 0.1 mM EGTA, Egg PA (200 μM), 20 mM MgCl_2_, 5.1 mM Triton-X100, 0.25 mM DTT, 50 μg protein, CTP (20 μM) and 2.5 μCi CTP [5′-^3^H]) (ARC #ART0343). For the assay, the subcellular fractions were suspended in ice-cold CDS buffer (50 mM Tris-HCL (pH 8.0), 50 mM KCl, 0.2 mM EGTA (ethylene glycol tetraacetic acid)) and 1/100 v/v protease inhibitor cocktail (Sigma, P8340). The PA was dried down under nitrogen and resuspended in the CDS assay buffer comprising of 125 mM Tris-HCl (pH 8), 250 mM KCl, 12.75 mM Triton X100, 5 mg/ml BSA and 0.625 mM DTT.

The components of the assay were added in the following order: 48 μl of protein sample (50 μg) (or bovine serum albumin (BSA) as a control protein), 40 μl PA suspension with 2 μl MgCl_2_ (1 M) added, and 10 μl of CTP (200 μM) mixed with 2.5 μCi CTP [5′-^3^H]. The reaction was incubated for 10 min at 30 °C. (We confirmed that the assay was linear with respect to protein concentration (0–200 μg) and time (0–12 min)). It was terminated with 375 μl acidified chloroform: methanol (1:2) and after phase separation with chloroform and water (125 μl each), the organic phase was recovered, washed with synthetic top phase, and the chloroform phase transferred to a scintillation vial. (The synthetic top phase was a mixture of methanol and water (1:0.9)). The chloroform was allowed to evaporate prior to counting the radioactivity in a scintillation counter. PI synthase activity was measured in a 100 μl assay containing 50 mM Tris-HCL (pH 7.4), 2 mM MnCl_2_, 50 μg protein sample, 20 μM unlabelled inositol and 2.5 μCi [^3^H]myo-inositol at 37 °C for 15 min. The reaction was conducted at 30 °C for 10 min and processed as above.

### 2.9 Subcellular fractionation of rat heart, brain and liver and H9c2 cells

Mitochondria from rat heart were prepared as described [37]. In brief, two hearts were transferred into TSE buffer (200 mM mannitol, 70 mM sucrose, 5 mM Tris-HCl (pH 7.4), and 2 mM EGTA), and perfused through the aorta briefly with 25 ml of cold TSE buffer to remove the blood. When treated with protease, the heart was further perfused with 5 ml of TSE buffer containing subtilisin (0.4 mg/ml) (Sigma P5380). The hearts were trimmed to remove non-ventricular tissue, cut into small fragments and homogenized with a motorized Potter Elvehjem glass homogenizer. All centrifugations were carried out at 4 °C. Nuclei and unbroken cells were pelleted at 850*g* (3000 rpm) for 5 min. The supernatant was decanted into a clean tube is referred to as whole cell lysate (WCL). Protease inhibitors (Cocktail I from Sigma P-8340) (1:100) were added and an aliquot retained. The mitochondria were pelleted at 9500*g* (11,000 rpm) for 15 min (Sorvall) and the supernatant retained to obtain the microsomes and cytosol. The mitochondrial pellet was gently washed with 2 ml of TSE buffer to remove a variable fluffy layer. The mitochondrial pellet was resuspended in 40 ml TSE buffer and homogenized in a glass homogenizer and recovered by centrifugation. This was repeated twice. Where indicated, the crude mitochondria were further purified on a Percoll gradient [38]. The Percoll solution was made by mixing 11.2 ml TSE buffer with 4.8 ml Percoll. The crude mitochondria were suspended in 12 ml of TSE buffer. The mitochondria (5 ml) were layered on 8 ml of Percoll solution and centrifuged in a swingout rotor at 27,000 rpm (95,000*g*) for 30 min. The mitochondria were recovered as well as the mitochondrial associated membranes (MAMs). The mitochondria were centrifuged and retained for subsequent analysis. The MAM fraction and the supernatant containing the cytosol and microsomes were centrifuged at 100,000*g* for 1 h to recover the MAM fraction, microsomes and cytosol. Protein concentrations in all the fractions were determined by BCA assay.

H9c2 cells were pelleted down and resuspended in 1 ml TSE buffer containing 1:100 dilution of protease inhibitors (Sigma). The cells were sonicated and centrifuged at 330*g* for 5 min at 4 °C to pellet unbroken cells. An aliquot of the supernatant (whole cell lysate, WCL) was retained. The remaining supernatant was centrifuged at 8200*g* for 10 min at 4 °C to pellet crude mitochondria. This was repeated twice, and the crude mitochondrial pellets combined, and washed with TSE buffer. The supernatant remaining after crude mitochondria had been sedimented, was centrifuged at 112000*g* at 4 °C for 1 h to pellet the microsomes. The crude mitochondrial pellet and the microsomal pellet was resuspended in CDS buffer and sonicated. Protein concentrations in all the fractions were determined by BCA assay. Cells from three T175 flasks (~ 2.4 × 10^7^ cells) were used for the fractionation per condition.

### 2.10 Removal of peripheral proteins with high salt and pH 11 treatment

Mitochondria used for these experiments were frozen at − 80 °C in order to compromise their integrity and thus gain access to the mitochondrial inner membranes. Mitochondria were centrifuged and the pellets suspended in either 0.2 M sodium bicarbonate (pH 11), 1 M NaCl (High salt) in CDS buffer (50 mM Tris-HCL (pH 8.0), 50 mM KCl, 0.2 mM EGTA) or CDS buffer. Each of the buffers contained 1:100 dilution v/v protease inhibitor cocktail (Sigma, P8340). After incubation of the sample on a rotating wheel at 4 °C for 60 min, the samples were recovered by centrifugation. Both the pellet and supernatant were analysed.

### 2.11 Measurement of cardiolipin levels

Proliferating H9c2 cell were seeded in 10 cm dishes (6.4 × 10^4^ cells/cm^2^) and transfected with a mixture of two TAMM41 siRNA (10 nM) with HiPerFect Transfection Reagent following the manufacturer instructions (Qiagen). At the same time, 1 μCi/ml ^14^C-acetate (Perkin Elmer) was added to the media to label the lipids to equilibrium. After 72 h, the cells were recovered by trypsinisation. The cells were sonicated and centrifuged at 330*g* for 5 min at 4 °C to pellet unbroken cells. The supernatant was spun at 8200*g* for 1 h at 4 °C to pellet crude mitochondria. The lipids were extracted, and analysed by HPTLC (Millipore) alongside lipid standards in the solvent mixture CHCl_3_ (75): MeOH (45): CH_3_COOH (3): H_2_O (1). The plates were then phosphorimaged for 3 days (^14^C) and read on a Fujifilm FLA-2000 before being analysed with AIDA software. Phosphatidylcholine was used as an internal standard, and the ratio of cardiolipin to phosphatidylcholine was used to determine the relative cardiolipin levels.

### 2.12 Statistical analysis

Statistical analysis was performed by using unpaired two-tailed *t*-test, or 1 or 2 way ANOVA with multiple comparisons analysing experimental data by means of the Prism 6 program (GraphPad software for Science, San Diego, CA USA).

## 3 Results

### 3.1 Differentiation of H9c2 cells with retinoic acid increases mitochondrial content, oxygen consumption and CDS1 immunoreactivity

**Fig. 1:**
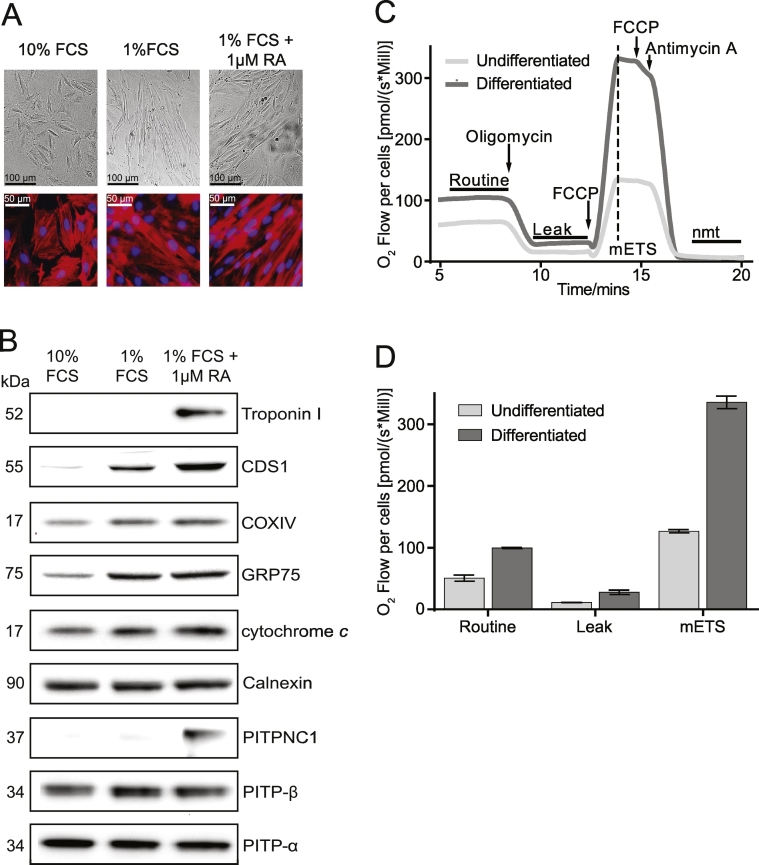
Differentiation of H9c2 cells with retinoic acid. [A] Morphology examined by Nomarski optics (upper panels) and cellular f-actin and nuclei stained with phalloidin and DAPI respectively (lower panels). [B] Western blot of cell lysates (50 μg protein) obtained from proliferating cells (10% FCS) and differentiated cells in either 1% FCS or 1% FCS with 1 μM retinoic acid (RA). [C] Example traces of oxygen consumption in proliferating and differentiated cells (1% FCS with RA); arrows indicate addition of drugs, and maximal ETS (electron transport system) indicated by a dotted line (nmt, non-mitochondrial). [D] Quantitation of the Routine, Leak and maximal ETS in proliferating and differentiated cells. The results are averages from two independent experiments for the differentiated cells (1% FCS with RA) and from three experiments for the proliferating cells. Error bars denote ± S.E.M.

Proliferating H9c2 myoblast cells are relatively undifferentiated phenotypically but can be induced to differentiate under reduced serum conditions. Skeletal or cardiac phenotypes can be induced depending on whether or not serum reduction is accompanied by daily treatment with all-*trans* retinoic acid [39], [40], [41], [42]. We induced differentiation by culturing H9c2 myoblasts in 1% fetal calf serum in the presence or absence of all-*trans*-retinoic acid for 8 days. Differentiation of H9c2 cells led to a pronounced change in morphology (Fig. 1A). The morphology of parental cells changed from being spindle to stellate shape with stress fibres to a large elongated shape occasionally multi-nucleated with the stress fibres being prominent (Fig. 1A). Cardiomyocyte-like differentiation was confirmed by the expression of troponin I (Fig. 1B). Although differentiation of H9c2 cells with 1% FCS was sufficient to induce increases in cytochrome *c* oxidase subunit IV (COXIV), GRP75 (75 kDa Glucose-regulated protein), and cytochrome *c* indicating an increase in mitochondrial biogenesis and/or functionality, retinoic acid treatment was required for expression of Troponin I. The increase in the expression of mitochondrial proteins was accompanied by an increase in oxygen consumption (Fig. 1C and D). Four components of the oxygen consumption trace are indicated (Fig. 1C), The Routine which measures the resting consumption, the Leak which is observed after inhibition of the ATPase with oligomycin, the maximal electron transport system (mETS) obtained in the presence of the uncoupler, FCCP and finally the non-mitochondrial (nmt) oxygen consumption obtained after addition of antimycin A (Fig. 1C). Compared to the undifferentiated cells, in the differentiated cells there was a robust increase in the Routine, Leak and mETS after subtraction of the nmt (Fig. 1C and D).

Retinoic acid increases the protein levels of the transcriptional coactivator PGC-1α (peroxisome proliferator-activated receptor γ coactivator-1α) [40], [42], a master regulator of mitochondrial phospholipid biogenesis [4], [5]. It has been recently reported that combined loss of PGC-1α and β from adult heart resulted in a decrease in cardiolipin levels and this was accompanied by reduced mRNA expression of CDS1 in mice [6]. We therefore examined whether CDS1 protein expression was increased during differentiation. A monoclonal antibody to CDS1 is commercially available and has been previously used for the detection of CDS1 [43], [44]. Using this antibody, a dramatic increase in a p55 protein running at the expected size for CDS1 (53 kDa) was observed upon differentiation. The increase was greatest when serum reduction was accompanied by the presence of retinoic acid (Fig. 1B).

We have previously shown that the PA/PI lipid transporter, PITPNC1 belonging to the PITP family is enriched in the heart [30] and we therefore examined its expression following differentiation of these cells. Proliferating H9c2 cells and H9c2 cells incubated with 1% FCS did not show significant protein expression of PITPNC1. However, differentiation with retinoic acid showed a pronounced increase in PITPNC1 levels (Fig. 1B). The increase in PITPNC1 was specific for this transfer protein, as no increases were observed in two other PITPs, PITPα and PITPβ (Fig. 1B).

### 3.2 Localisation of CDS1 immunoreactivity and CDS activity with a mitochondrial fraction

**Fig. 2:**
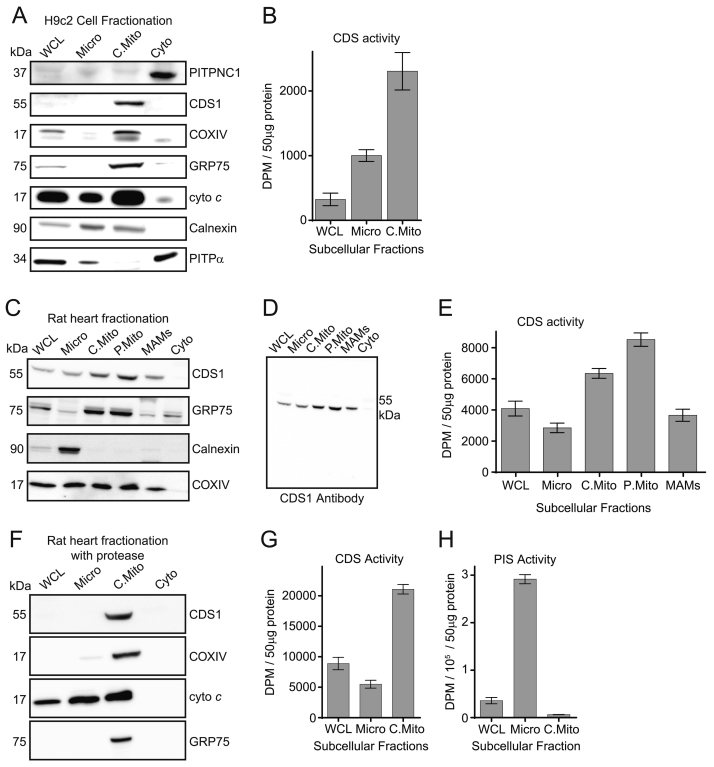
Localisation of CDS1 immunoreactivity and CDS activity to mitochondria. [A, B] Differentiated H9c2 cells fractionated and analysed for [A] CDS1 immunoreactivity by Western blot; [B] CDS activity. [C-E] Rat heart fractionated and analysed for [C] CDS1 immunoreactivity and markers, [D] CDS1 immunoreactivity (entire Western blot shown) and [E] CDS activity. [F–H] Rat heart fractionated in the presence of the protease, subtilisin and analysed for [F] CDS1 immunoreactivity by western blot, [G] CDS activity and [H] PI synthase (PIS) activity. CDS and PIS activity was monitored in triplicate and error bars denote ± S.E.M. COXIV, GRP75 and cyto *c* are markers for mitochondria, PITPNC1 and PITPα are cytosolic markers and calnexin is a marker for the ER. WCL, whole cell lysate; Micro, microsomes; C.Mito, crude mitochondria; Cyto, cytosol; P.Mito, pure mitochondria, MAMs, mitochondrial associated membranes; PIS, PI synthase; CDS, CDP-diacylglycerol synthase.

Having observed an increase in CDS1 and PITPNC1 protein upon differentiation, we next examined the subcellular localisation of these proteins. Differentiated H9c2 cells were fractionated into microsomes, crude mitochondria and cytosol. CDS1 immunoreactivity was found mainly in the mitochondrial fraction as indicated by the presence of COXIV, GRP75 and cytochrome *c* (cyto *c*) (Fig. 2A). The subcellular fractions were also analysed for CDS activity and mitochondria were found to be highly enriched (Fig. 2B). The PITP proteins, PITPα and PITPNC1, localised mainly to the cytosol fraction. Since some calnexin was also found in the mitochondrial fraction, it suggested that CDS1 could be associated within an ER compartment which was tightly associated with the mitochondria. Mitochondria normally co-purify with the sub-region of the ER, referred to as mitochondria-associated endoplasmic reticulum membranes (MAMs) [45]. To analyse this further, we used rat heart due to availability of larger amounts of tissue. Again, we found that CDS1 immunoreactivity was mainly associated with the crude mitochondrial fraction (Fig. 2C). The crude mitochondrial fraction was further purified on Percoll gradients to obtain pure mitochondria and CDS1 immunoreactivity was still present in this fraction (Fig. 2C). (The entire blot for CDS1 immunoreactivity is shown in Fig. 2D to demonstrate that the antibody recognises a single band on the blot.) We also monitored CDS enzyme activity and found that although all fractions contain CDS activity, the highest activity was present in the pure mitochondrial fraction (Fig. 2E).

In the heart, two populations of mitochondria have been identified, mitochondria that lie beneath the sarcolemma and another population that lie between the myofibrils. To release the inter-fibrillar mitochondria, a protease is often included during mitochondrial purification [2]. We therefore purified the mitochondria in the presence of the protease. Analysis of the fractions indicated that CDS1 immunoreactivity was still found in the mitochondrial fraction (Fig. 2F). When the fractions were analysed by SDS-PAGE, it was noted that many of the high molecular weight proteins had disappeared from the cytosolic and microsomal fraction but not in the mitochondrial fraction due to the presence of the protease. Nonetheless, the CDS1 immunoreactive protein and CDS activity were preserved in the mitochondrial fraction suggesting protection from the protease (Fig. 2F and G). We also monitored PI synthase activity and found that its activity was also preserved and was mainly present in the microsomes (Fig. 2H). PI synthase is a small integral protein of 21 kDa and does not seem to be affected by the presence of the protease.

**Fig. 3:**
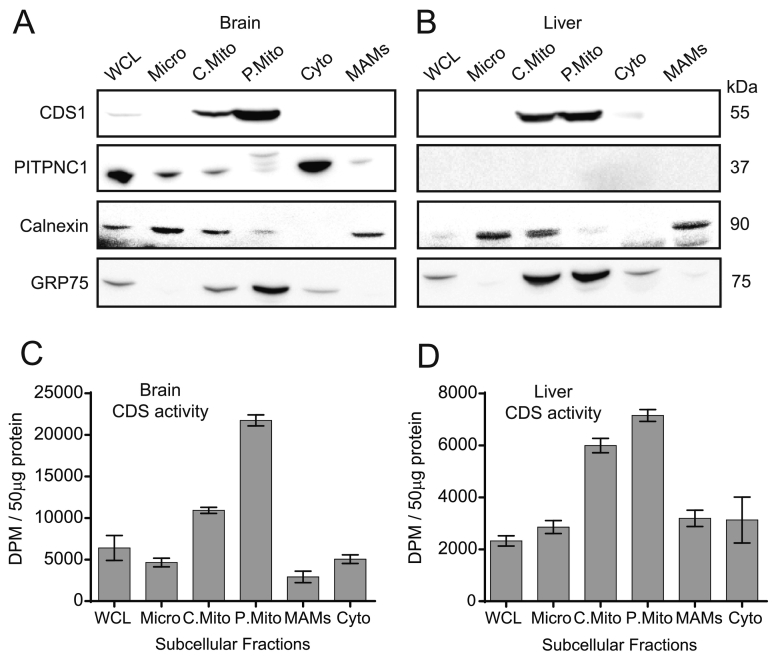
CDS1 immunoreactivity and CDS activity localises to mitochondria in rat brain and liver. [A, C] Rat brain and [B, D] liver fractionated and analysed by CDS1 immunoreactivity by Western blot and for CDS activity. CDS activity was monitored in triplicate and error bars denote S.E.M. WCL, whole cell lysate; Micro, microsomes; C.Mito, crude mitochondria; Cyto, cytosol; P.Mito, pure mitochondria, MAMs, mitochondrial associated membranes; CDS, CDP-diacylglycerol synthase.

To examine whether the localisation of CDS1 immunoreactivity and enzyme activity is also associated with mitochondria in other tissues, we tested both rat brain and liver (Fig. 3). Both tissues were fractionated into microsomes, mitochondria and cytosol. The crude mitochondria were further purified on a Percoll gradient to obtain pure mitochondria and the MAMs. In both brain and liver, CDS1 immunoreactivity was exclusively associated with the pure mitochondrial fraction (Fig. 3A, B). We also monitored CDS activity and found that the mitochondria had the highest amount of activity in both brain and liver (Fig. 3C, D). The presence of PITPNC1 was also monitored and whilst brain was highly enriched, none was detectable in liver.

### 3.3 Differentiation of H9c2 cells did not lead to an increase in CDS1 mRNA

**Fig. 4:**
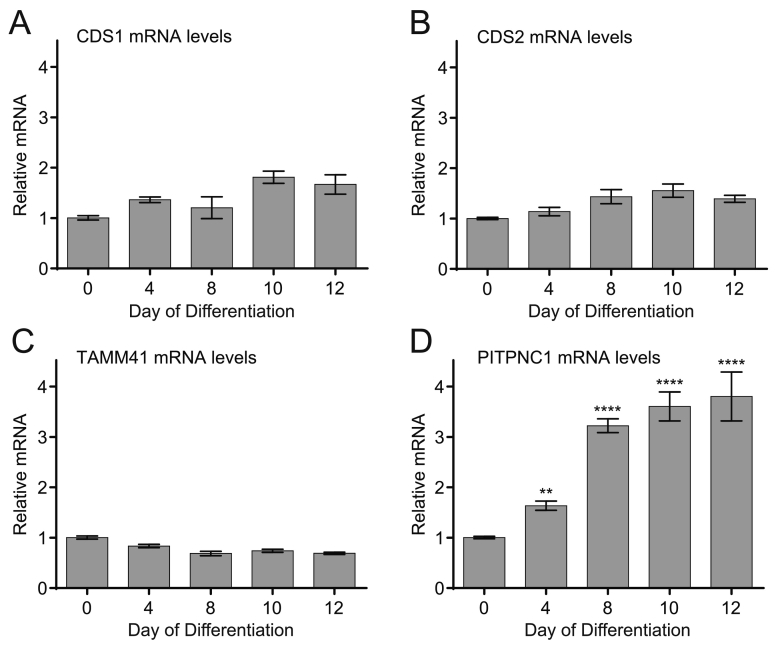
Differentiation of H9c2 cells does not cause an increase in CDS1 mRNA expression. H9c2 cells were differentiated with retinoic acid and samples removed daily to monitor mRNA levels. [A] CDS1 mRNA; [B] CDS2 mRNA; [C] TAMM41 mRNA; [D] PITPNC1 mRNA. Data from 3 experiments done in triplicate ± S.E.M.

To complement the increase in CDS1 and PITPNC1 immunoreactivity following differentiation, we examined mRNA for CDS1, CDS2, TAMM41 and PITPNC1. TAMM41 is the mammalian homologue of the recently identified CDS enzyme in the yeast, *S. cerevisiae* [26] which is unrelated to CDS1 and 2. No significant changes in CDS1, CDS2 or TAMM41 mRNA was observed (Fig. 4A–C). This result was surprising considering the dramatic change observed in CDS1 immunoreactivity (Fig. 1). In contrast to CDS1 mRNA, an increase in PITPNC1 mRNA was observed upon differentiation (Fig. 4D).

### 3.4 Characterization of the CDS1 antibody

The localisation of CDS1 immunoreactivity in mitochondria was unexpected as several studies had shown that CDS1 when expressed as a tagged protein localises at the ER [7], [18], [19]. The CDS1 monoclonal antibody used here is a commercial antibody and although used in two previous studies has not been adequately characterised [43], [44]. For characterization of the antibody, we asked whether the antibody recognises CDS1 and was specific i.e. it does not cross react with CDS2. We also used siRNA to knockdown CDS1 to monitor the disappearance of the CDS1 immunoreactivity.

**Fig. 5:**
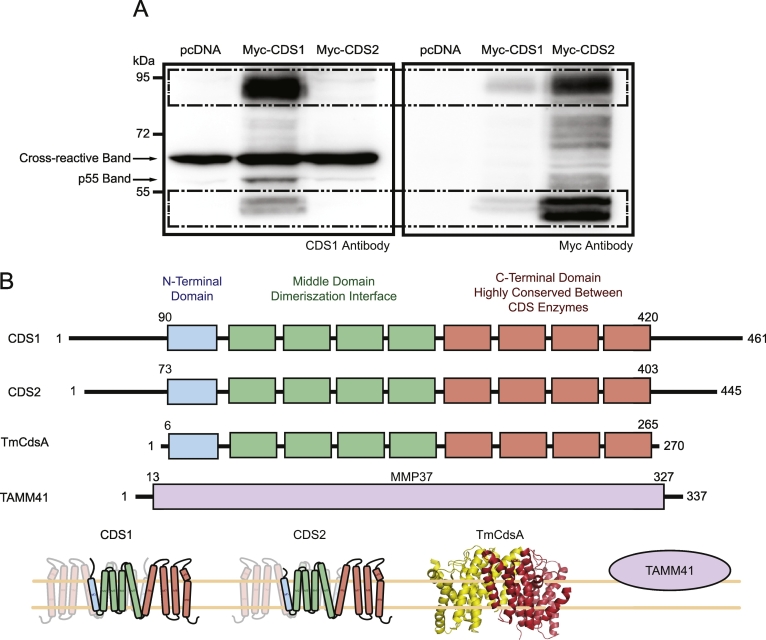
CDS1 antibody recognises over-expressed CDS1 but not over-expressed CDS2. [A] Myc-tagged CDS1 and CDS2 were expressed in COS-7 cells and membranes obtained after centrifugation. Membranes (25 μg protein per lane) probed with antibodies to CDS1 (left panel) and Myc (right panel). The boxed region highlights the over-expressed proteins. CDS proteins run as monomers (~ 45 kDa) and as dimers (~ 95 kDa). The CDS1 antibody (Abcam) also recognises a p55 protein in the CDS1-over-expressing cells. [B] Topological organisation of the CDS enzymes, CDS1, CDS2 and TmCdsA based on the structure of TmCdsA, and the structurally unrelated CDS enzyme, TAMM41. The transmembrane domains of CDS1 and CDS2 are shown in coloured boxes. Blue boxes, N-terminal domain, green boxes, middle domain which is the dimerization interface and the orange boxes which is the highly-conserved C-terminal domain. Also shown is the three dimensional structure of TmCdsA which forms a dimer; the monomers are coloured red or yellow. Cartoon representation of CDS1 and CDS2 based on TmCdsA dimer. TAMM41 (NP_001271330.1) is shown as a peripheral membrane protein containing the MMP37 domain (MMP37, mitochondrial matrix proteins of 37 kDa; PFAM: 09139) [46].

We expressed Myc-tagged CDS1 and CDS2 in COS-7 cells and after cell homogenisation, membranes were obtained which were analysed by western blot with either the CDS1 or the Myc antibody. (We also examined a commercial CDS2 antibody but it failed to detect over-expressed CDS2). The CDS1 and the Myc antibody recognised two common bands, a doublet at ~ 45 kDa and at ~ 95 kDa in the Myc-CDS1-expressing cells (Fig. 5A, marked with a box with dotted lines). We also observed that the CDS1 antibody recognised an additional band at around 55 kDa only in the CDS1-over-expressing cells. (We stress that this immunoreactive band is induced by CDS1 over-expression and is the same size as the immunoreactive band observed in Fig. 1, Fig. 2, Fig. 3 suggesting that it is likely to be the same cross-reactive protein). The CDS1 antibody did not recognise CDS2 although it was robustly expressed as confirmed with the Myc antibody (Fig. 5A, right panel). Like CDS1, CDS2 was detected as two bands, a doublet at ~ 45 kDa and at ~ 95 kDa. Expression of the CDS proteins was also confirmed by activity measurements of the COS-7 membranes (see Fig. 7D). Although CDS2 expression was much greater than CDS1 as observed in the Myc-stained blot, the CDS activity did not match the expression profile. We attribute this to the PA substrate used for the assay. We have used egg PA which is not a good substrate for CDS2 [19]. CDS2 prefers *sn-*1-stearoyl (C18:0), *sn-2* arachidonyl (C20:4)-PA unlike CDS1 which prefers mono-saturated fatty acids as used here [19].

An anomaly is immediately apparent; a substantial fraction of the over-expressed CDS1 and CDS2 runs as a ~ 95 kDa protein on an SDS-PAGE gel (theoretical molecular weight is ~ 53 kDa) suggesting that some of the CDS proteins remain as a dimer despite the presence of SDS. This observation concurs with the structure of Cds from the bacterium, *Thermotoga maritama* (TmCdsA) which has been recently reported to form a dimer [17] (Fig. 5B). Our attempts to dissociate the dimer further were unsuccessful.

**Fig. 6:**
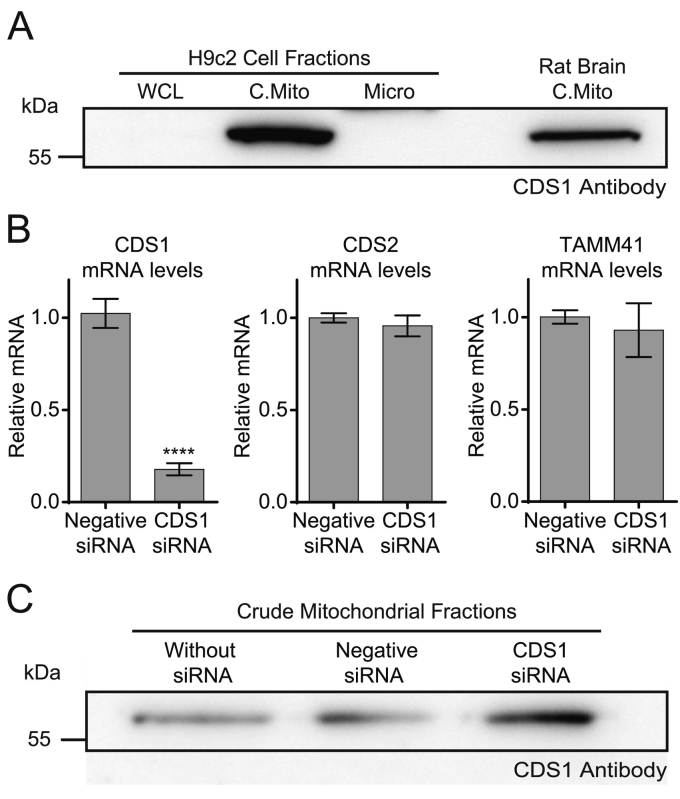
Knockdown of CDS1 with siRNA does not lead to a reduction in the p55 band. [A] Detection of the p55 band with the CDS1 antibody in undifferentiated H9c2 cells is only possible when mitochondrial fractions are prepared. [B] Validation of CDS1 siRNA. A decrease in CDS1 mRNA (P < 0.0001, n = 6, ± S.E.M.) but not CDS2 or TAMM41 is observed when undifferentiated H9c2 cells were treated with CDS1 siRNA. [C] Mitochondrial fractions prepared from control, negative siRNA-treated and CDS1 siRNA-treated H9c2 cells were analysed for CDS1 immunoreactivity by Western blot. WCL, whole cell lysate; C.Mito, crude mitochondria; Micro, microsomes.

In mitochondria prepared from either heart, liver or brain, the CDS1 antibody recognised a single band running at 55 kDa (Figs. 2D, 3A and B). These data suggest that the CDS1 antibody, although able to detect over-expressed CDS1, is very likely detecting a cross-reactive p55 mitochondrial protein. Further support for this suggestion comes from studies with siRNA. The p55 protein is induced sufficiently for detection in whole cell lysates following differentiation of H9c2 cells (Fig. 1B). Unfortunately, it was found that siRNA transfection during differentiation frequently led to inhibition of the differentiation process, as no increase in Troponin I, PITPNC1 or the p55 protein (detected with the CDS1 antibody) was apparent (data not shown). The disruption of the differentiation process was observed even with a negative control siRNA and was therefore a non-specific consequence of transfection during differentiation. Thus undifferentiated H9c2 cells had to be used for the siRNA experiments. Since the immunoreactivity detected with the CDS1 antibody in undifferentiated H9c2 cell lysates is very weak (Fig. 1B and Fig. 6A), we prepared mitochondrial fractions to enrich the signal; the p55 band was now readily detected (Fig. 6A). The siRNA used to knockdown CDS1 was tested on the mRNA levels (Fig. 6B). RT-qPCR confirmed that the siRNA reduced CDS1 mRNA with no change in CDS2 or TAMM41 mRNA (Fig. 6B). Comparison of mitochondria isolated from control and CDS1-siRNA-treated H9c2 cells showed that the band at 55 kDa was resistant to CDS1 siRNA treatment (Fig. 6C) further supporting the notion that this is a cross-reactive band detected by the CDS1 antibody and not CDS1.

### 3.5 Mitochondrial CDS activity is due to a peripheral protein, TAMM41

**Fig. 7:**
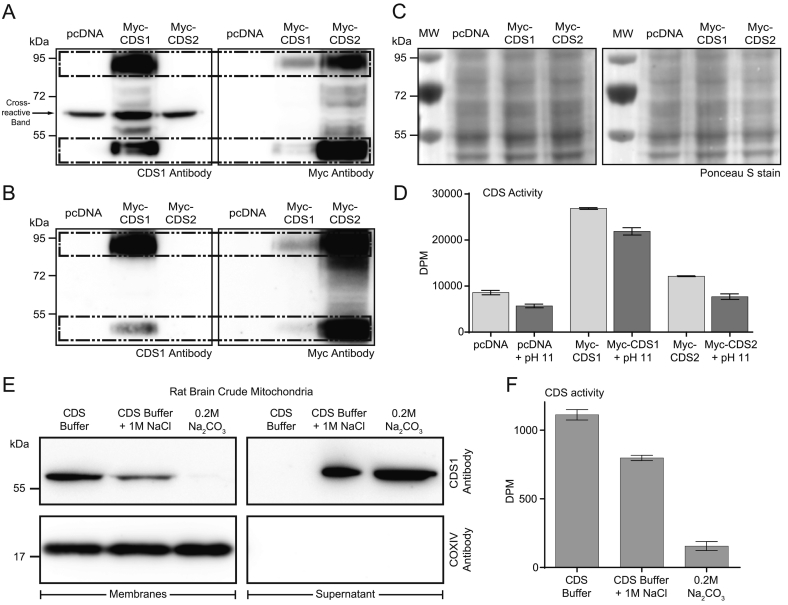
Mitochondrial CDS activity is due to a peripheral membrane protein. [A, B] Total membranes (50 μg protein) prepared from COS-7 cells expressing either Myc-CDS1 or Myc-CDS2 were analysed by western blot [A] before and [B] after treatment with 0.2 M sodium bicarbonate (pH 11). [C] Loading Control: Ponceau S stain of blots in [A]. [D] CDS activity of COS-7 cell total membranes (50 μg protein) before and after treatment with sodium bicarbonate. [E, F] Mitochondria purified from rat brain were incubated with high salt (1 M NaCl) or with sodium bicarbonate to remove peripheral proteins. [E] The residual mitochondrial membranes and supernatant were analysed by Western blot with CDS1 and COXIV antibody. [F] The membranes were analysed for CDS activity.

Since the CDS1 antibody was only detecting the cross-reactive band in tissue samples (see Fig. 2D), a different approach was taken to examine whether CDS1 was responsible for the mitochondrial activity. We first over-expressed Myc-tagged CDS1 and CDS2 in COS-7 cells and prepared total membranes from the lysates. The total membranes were analysed by Western blot before and after treatment with 0.2 M sodium bicarbonate (pH 11) to remove peripherally-associated proteins. As expected, CDS1 and CDS2 remained membrane-associated (compare Fig. 7A with 7B) as CDS enzymes are integral membrane proteins [17] (see also Fig. 5B). However, several proteins were removed by this treatment including the p55 band that is specifically observed upon CDS1 over-expression. To check whether CDS1 and CDS2 activity were affected by exposure to pH 11, we also monitored CDS activity; only a small decrease in CDS activity was observed in membranes exposed to pH 11 suggesting that this treatment does not affect CDS1 or 2 activities (Fig. 7D).

Having confirmed that CDS1 is an integral membrane protein and its activity is unaffected when membranes are treated to remove peripheral proteins, we used mitochondria to examine the nature of the CDS activity as well as the p55 band. We incubated the crude mitochondria with high salt (1 M NaCl in CDS buffer) or a sodium bicarbonate buffer (0.2 M, pH 11). The residual membranes and the resultant supernatant were recovered by centrifugation and the supernatant concentrated and analysed for CDS1 immunoreactivity. The p55 protein was removed from the membranes and recovered in the supernatant indicating that it is a peripheral membrane protein (Fig. 7E). These data conclusively demonstrates that the CDS1 antibody, although able to detect the over-expressed CDS1 protein by western blot, also recognises an additional peripheral protein that is exclusively localised in mitochondria.

**Fig. 8:**
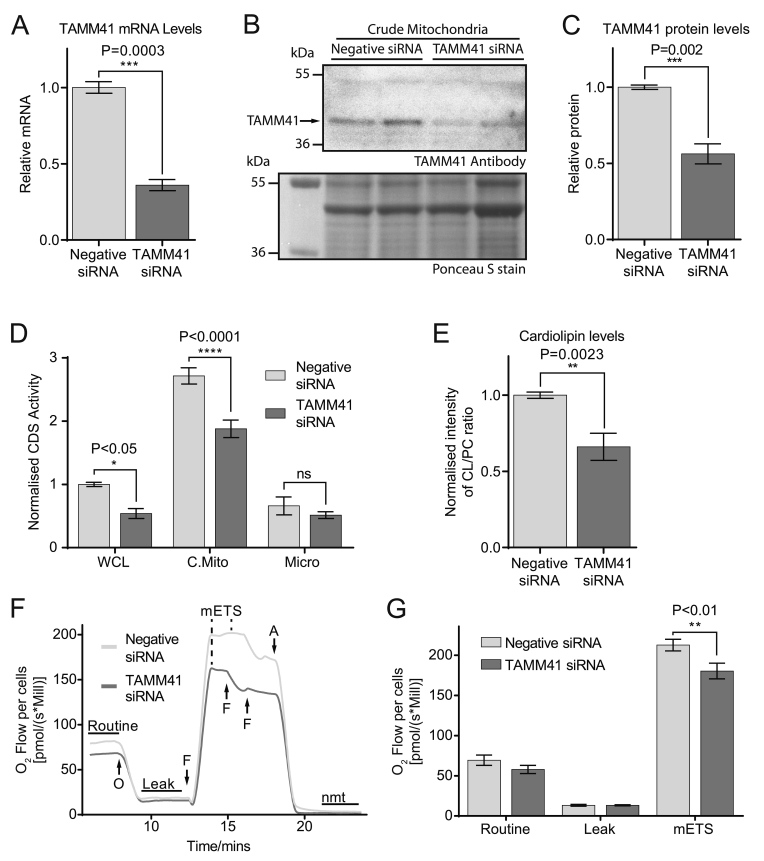
Mitochondrial CDS activity is due to TAMM41 and its knockdown reduces oxygen consumption. [A] Knockdown of TAMM1 mRNA by siRNA in H9c2 cells (n = 3, unpaired two-tailed *t*-test). [B] Western blot showing knockdown of TAMM41 protein in the crude mitochondrial fractions from TAMM41 knockdown cells. Loading control: Ponceau S stain of the blot. Crude mitochondria were prepared from H9c2 cells treated with control or TAMM41 siRNA. Representative blot of two independent crude mitochondrial fractions. [C] Quantification of TAMM41 protein in crude mitochondria from 4 independent fractionations (n = 4, unpaired two-tailed *t*-test). [D] CDS activity after TAMM41 knockdown in H9c2 subcellular fractions. Results are from two independent experiments done in triplicate (n = 6, two-way ANOVA with multiple comparisons). The average activity in control whole cell lysates from different experiments was in the range of 2–6 pmol/mg/min. WCL, whole cell lysate; C.Mito, crude mitochondria; Micro, microsomes; [E] Cardiolipin levels in crude mitochondria prepared from control and TAMM41 siRNA-treated H9c2 cells. H9c2 cells were labelled to near equilibrium with ^14^C-acetate for 72 h and lipids extracted from crude mitochondrial fractions and analysed by TLC. Results are from 3 independent experiments done in duplicate or triplicate (n = 8, unpaired two-tailed *t*-test). [F] Example trace of oxygen consumption in control and TAMM41 knockdown H9c2 cells; arrows indicate addition of drugs and maximal ETS (electron transport system) shown by dotted line. nmt, non-mitochondrial; O, oligomycin; F, FCCP; A, antimycin A. [G] Quantitation of the Routine, Leak and maximal ETS in control and TAMM41-knockdown cells. The results are averages from three independent experiments; two way ANOVA with multiple comparisons Error bars denote ± S.E.M.

To examine the nature of the mitochondrial CDS activity, the mitochondria stripped of peripheral proteins were assessed for CDS activity. The mitochondrial activity was partially lost after treatment with NaCl or near-completely lost after treatment with sodium bicarbonate (Fig. 7F) excluding this activity to be due to CDS1 or CDS2. We therefore surmised that the CDS activity present in mitochondria could potentially be due to the mammalian homologue of the yeast enzyme, Tam41 (also known as MMP37, mitochondrial matrix protein of 37 kDa [46], [47]). To test this, H9c2 cells were treated with TAMM41 siRNA. We first confirmed that the siRNA was effective in reducing the expression of TAMM41 mRNA (Fig. 8A). We have used an anti-TAMM41 antibody from ATLAS antibodies (HPA036834) and first validated the antibody. Crude mitochondria prepared from H9c2 cells had to be used to enrich the antigen, and a band of the appropriate molecular weight was observed. This band was reduced in the TAMM41 knockdown crude mitochondria (Fig. 8B and C).

H9c2 cells were fractionated to separate mitochondria from the microsomes and fractions analysed for CDS activity. A significant reduction was observed in the mitochondrial fraction prepared from cells treated with TAMM41 siRNA (Fig. 8D). These data confirm that the activity observed in mitochondria is due to TAMM41. To assess whether knockdown of TAMM41 resulted in changes in cardiolipin level, H9c2 cells were grown in ^14^C-acetate for 72 h to label the lipids to near equilibrium. Mitochondria were prepared from control and knockdown cells and analysed by TLC. A significant decrease in cardiolipin was observed (Fig. 8E).

Finally, we examined whether the knockdown of TAMM41 had any impact on respiration as cardiolipin has numerous roles in the stabilisation of the COX complexes and other respiratory roles [48]. Fig. 8F shows an example trace of control and TAMM41 knockdown cells and Fig. 8G shows the oxygen flow per million cells across 3 experiments. Over the course of the experiments there were variations in the routine reading – the basal oxygen consumption of the cells. Although there was no statistical significance observed in the three combined experiments, throughout each experiment there was a downward trend; the TAMM41 knockdown cells had a lower basal level of oxygen consumption (Negative siRNA 69.3 ± 6.4 pmol/(s*million cells); TAMM41 siRNA, 57.8 ± 5.1 (pmol/s*million cells)). When looking at the maximal capacity of the electron transport chain (mETS), the mETS of the TAMM41 knockdown had significantly decreased from 212.6 ± 7.2 to 180.3 ± 9.8 pmol/(s*million cells) (p < 0.01).

**Fig. 9:**
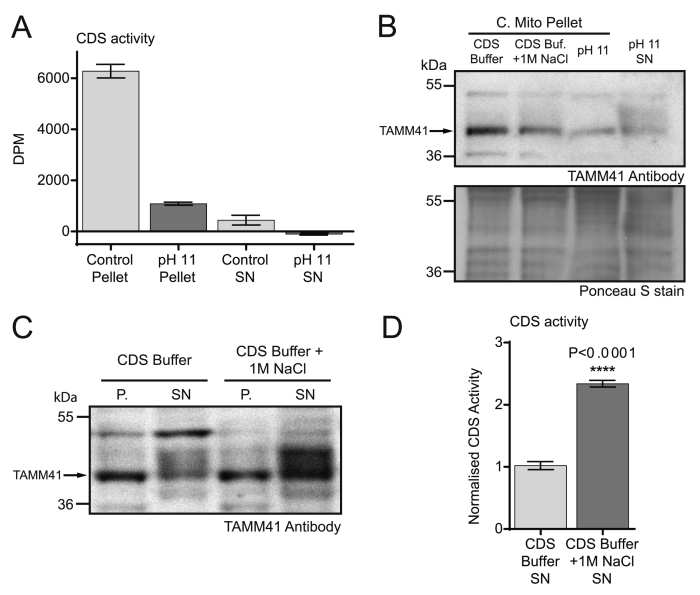
TAMM41 protein is released from the mitochondria after treatment with high salt and its activity is recovered in the supernatant. [A] Liver mitochondria were incubated with CDS buffer (50 mM Tris-HCL (pH 8.0), 50 mM KCl, 0.2 mM EGTA) or with 0.2 M sodium bicarbonate (pH 11). The supernatants were recovered, concentrated and buffer exchanged. CDS activity was monitored in the pellets and the supernatants. [B] Western blot of crude mitochondria treated with 1 M NaCl and pH 11 buffer and pH 11 supernatant. [C] Liver mitochondria were incubated with control buffer or control buffer supplemented with 1 M sodium chloride for 1 h. The mitochondrial pellets and the respective supernatants were Western blotted with TAMM41 antibody. P., pellet; SN, supernatant. [D] The supernatant from [C] was concentrated, buffer exchanged and CDS activity was assessed. Data from two experiments performed in triplicate ± S.E.M.

Treatment of mitochondrial membranes with pH 11 buffer led to a substantial loss of CDS activity (Fig. 7F). We therefore wanted to examine whether the activity could be recovered in the supernatant. Rat brain mitochondria were incubated with CDS buffer (50 mM Tris-HCL (pH 8.0), 50 mM KCl, 0.2 mM EGTA) or with 0.2 M sodium bicarbonate (pH 11). The supernatants were recovered and concentrated and examined for CDS activity alongside the mitochondrial pellets (Fig. 9A). As expected the mitochondria recovered after pH 11 treatment had lost a substantial amount of activity. From three independent experiments, the residual activity left was between 5 and 15%. A small amount of activity was recovered in the control supernatant but none in the pH 11 supernatant (Fig. 9A). (The supernatants were concentrated and buffer exchanged before analysis). This result suggested that prior exposure to pH 11 irreversibly inactivates TAMM41 activity.

Since 1 M salt treatment of mitochondria also shows some reduction in CDS activity (Fig. 7F), we examined whether CDS activity could be recovered from the supernatant after treatment with 1 M NaCl. We first used the antibody to demonstrate that the TAMM41 protein could be removed from the mitochondria with either salt treatment or with pH 11. As expected salt treatment removed less protein compared to pH 11 treatment. Moreover, we recovered TAMM41 protein in the pH 11 supernatant (Fig. 9B).

Crude mitochondria were incubated with high salt or CDS buffer. The supernatant and the recovered pellets were examined for TAMM41 immunoreactivity (Fig. 9C). We noted that control supernatant contained some TAMM41 immunoreactivity which increased substantially in the supernatant obtained after salt treatment. The supernatants derived after treatment were concentrated and buffer exchanged to remove the high salt. Measurement of activity in these two supernatants showed that the salt extract had greater activity compared to control (Fig. 9D).

### 3.6 CDS1 and CDS2 make a minor contribution to crude mitochondrial CDS activity

**Fig. 10:**
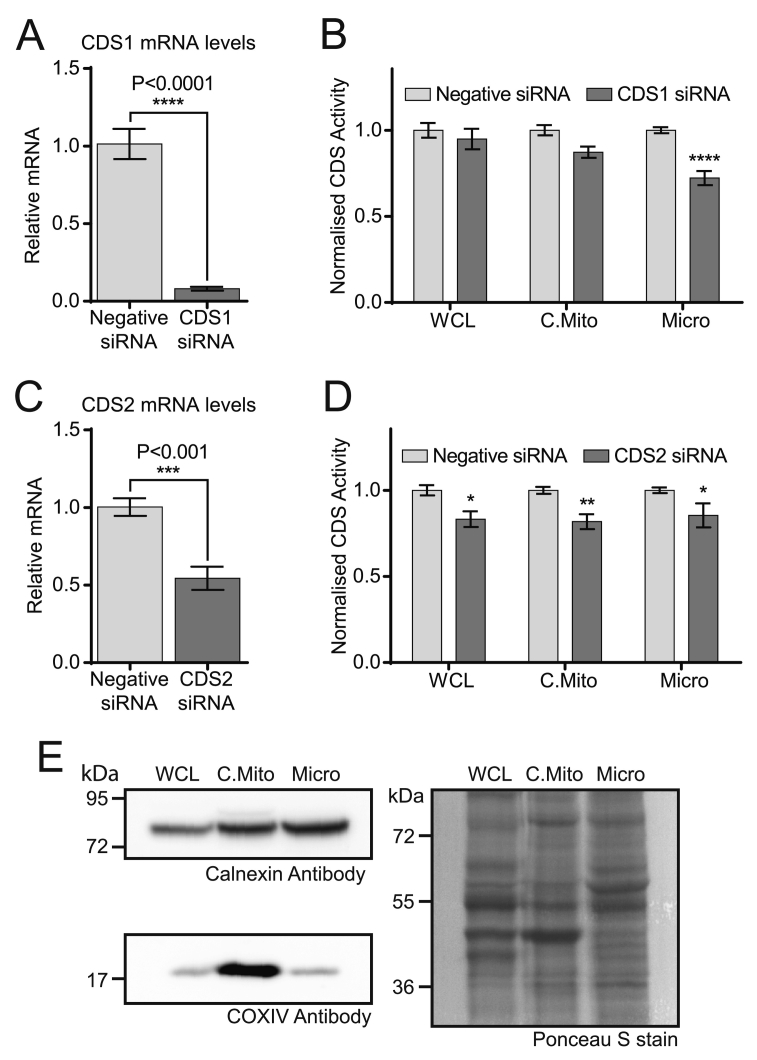
Crude mitochondria contain residual CDS1 and CDS2 activity that can be accounted for by ER associated membranes. [A, C] Knockdown of [A] CDS1 mRNA and [C] CDS2 mRNA by siRNA in H9c2 cells. [B, D] CDS activity after [B] CDS1 knockdown and [D] CDS2 knockdown in H9c2 subcellular fractions. Results from three independent experiments in triplicate were combined. In each experiment, the control sample was set at 1 and the knockdown samples are a proportion of the control. [E] Subcellular fractions (WCL, crude mitochondria and microsomes) analysed by Western blot for calnexin (ER marker) and COXIV (mitochondrial marker). A representative blot is shown from four separate fractionations.

In TAMM41 knockdown H9c2 cells, a substantial fraction of CDS activity was still present in mitochondria (Fig. 8D); this could be accounted for by incomplete knockdown (Fig. 8B and C). However, some CDS activity is retained after treatment of mitochondria with pH 11 which has to be contributed by CDS1 or CDS2. To examine this contribution, we used siRNA to knockdown CDS1 and CDS2 mRNA (Fig. 10A and C). The H9c2 cells from control and siRNA treated cells were fractionated and CDS activity monitored (Fig. 10B and D). We observed a slight decrease in mitochondrial CDS activity in both CDS1 and CDS2 knockdown cells. Since crude mitochondria were used, we also monitored the presence of ER membranes in the crude mitochondria. As expected, the crude mitochondria contained significant ER membranes (Fig. 10E).

## 4 Discussion

### 4.1 TAMM41 accounts for the mitochondrial CDS activity

The most important finding of this study is the identification of the mitochondrial CDS activity as TAMM41 and not CDS1. Previous studies had indicated that CDS activity was associated with mitochondria as well as with microsomes and yet expression of the two CDS proteins was normally found in the ER [7], [19], leaving open the question of the identity of the mitochondrial CDS activity. Fractionation of mitochondria from H9c2 cells and from tissues including rat heart, liver and brain indicated that purified mitochondria are highly enriched in CDS activity. Although using a commercial antibody suggested that this was due to CDS1, we provide compelling evidence that this activity is not due to CDS1 but due to a peripheral protein, TAMM41. Recent studies in yeast have identified a new enzyme, Tam41 that possesses CDS activity. Tam41 is a peripheral membrane protein that localises in the inner mitochondrial matrix where it is involved in cardiolipin synthesis [26]. Tam41 and the yeast Cds1 have different evolutionary origins and are not related by sequence although both possess CDS activity [26] (See Fig. 5B). A mammalian homologue of Tam41, TAMM41 (alias C3orf31) is a protein-coding gene present in the NCBI database and in the human MitoCarta2 database. It comprises of 337 amino acid residues with a calculated molecular weight of 35 kDa and possesses a mitochondrial targeting sequence. Mitochondrial CDS activity (Fig. 8D) and cardiolipin levels (Fig. 8E) were partially reduced when H9c2 cells were treated with TAMM41 siRNA. Cardiolipin is an essential lipid for the activity of a number of key mitochondrial enzymes involved in cellular energy metabolism. Consequently, a modest decrease in basal oxygen consumption as well as in the maximal capacity of the electron transport chain was observed (Fig. 8F, G).

CDS activity is only partially reduced in TAMM41 knockdown mitochondria. This reduction is probably partial due to incomplete knockdown of TAMM41. Alternatively, CDS1 or CDS2 could contribute to mitochondrial activity. Our data excludes this possibility; the main evidence comes from the observation that only 5–15% of CDS activity remains in mitochondria after TAMM41 is irreversibly inactivated by pH 11 exposure. To identify whether the residual activity is due to CDS1 or CDS2, both CDS enzymes were knocked-down by siRNA. Knockdown of CDS2, but not CDS1, lead to a small decrease in crude mitochondrial CDS activity (Fig. 10B and D). However, the crude mitochondria were found to be associated with ER (Fig. 10E), and therefore the CDS2 activity is likely derived from that compartment. We conclude that the CDS activity in mitochondria is exclusively due to TAMM41 and the ER membranes associated with mitochondria are responsible for the residual CDS activity.

### 4.2 The CDS1 antibody recognises a cross-reactive p55 protein

In this study, we used a commercial antibody to examine for CDS1 protein previously used in two other publications [43], [44]. In these publications as well as our study, this monoclonal antibody recognised a p55 protein. Indeed as shown in Fig. 2D, the antibody only recognises a single band on the gel when rat heart, liver or brain fractions were analysed. Because of our unexpected results indicating that CDS1 was a mitochondrial protein, we characterised the antibody. It appears to be normal practice that commercial antibodies are often used without any further authentication. We validated the antibody using over-expressed CDS1 and found that the antibody did recognise CDS1 but not CDS2 by western blot (Fig. 5A). However, the antibody only detects a cross-reactive p55 mitochondrial protein in heart, liver or brain. In COS-7 cells, the antibody also detects a p55 protein which is induced when CDS1 is over-expressed (Figs. 5A and 7A). In H9c2 cells the protein is present at low levels and is upregulated following differentiation to either skeletal muscle or cardiomyocytes (Fig. 1B). These data suggest that CDS1 over-expression does regulate mitochondrial dynamics by upregulating a mitochondrial protein.

Our attempts to identify this cross-reactive protein have not been successful. We partially purified the protein from mitochondria and the p55 protein band was analysed by Mass Spectroscopy. Although several promising hits were obtained including thioredoxin reductase 2 (TXNRD2), none of these hits were recognised by the CDS1 antibody. Hence, we were unable to confirm the identity of the p55 protein. The identity of this protein would be interesting for the following reasons: Firstly, differentiation of H9c2 cells causes a dramatic increase in its expression. Secondly, this antibody has been used in two other studies and we speculate that the protein identified in these two studies was not CDS1 but the p55 protein identified here. In one of these studies, the panel of non-small cell lung cancer (NSCLC) cell lines were examined for genes that were up-regulated by the transcription factor, ZEB1. CDS1 mRNA was found to be lower in cells that had high levels of ZEB1 mRNA and vice versa. These results were validated by showing that over-expression of ZEB1 in H358 cells caused a considerable decrease in CDS1 mRNA whilst siRNA for ZEB1 (and ZEB2) led to a considerable increase in CDS1 mRNA. In addition to monitoring mRNA, they used the same commercial monoclonal CDS1 antibody used here to confirm their data. They showed that the antibody recognised a p55 protein; we would suggest that the immunoreactive band is unlikely to be CDS1 but a mitochondrial protein [43]. Thus ZEB1 appears to downregulate not only CDS1 (shown by mRNA) but also a mitochondrial protein (shown by western blot). The ZEB family of zinc finger transcription factors are essential players during normal embryonic development and one characteristic is that they induce epithelial to mesenchymal transition (EMT), a process that reorganizes epithelial cells to become migratory mesenchymal cells. The relevance of ZEB1 and mitochondria might be an interesting topic for exploration. This study shows a relationship between CDS1 mRNA and the p55 band; a decrease in CDS1 mRNA correlated with the decrease in the p55 band. In our study we observe the converse; over-expression of CDS1 increased the expression of the p55 protein (Figs. 5A and 7A). Thus there appears to be a strong link between CDS1 and the p55 mitochondrial protein.

### 4.3 Criteria for antibody characterization

Since commercial antibodies are the most commonly used tools in the biosciences, it is important that all antibodies should be validated prior to use. The use of uncharacterised antibodies in the scientific literature is very common and this may compromise these studies. This issue has been highlighted recently [49], [50], [51] and here we provide an example of a previously-used commercial antibody that predominantly recognises a cross-reactive protein rather than the target protein. We suggest the following minimum criteria that should be applied routinely for validation of new antibodies. These criteria should be applied for every new technique that the antibody is used for. Thus an antibody validated for western blot should be independently validated if used for immunofluorescence. Our three criteria for western blot are: [1] the antibody should recognise over-expressed protein or recombinant protein; [2] the antibody should recognise the protein of the expected size; [3] knockout of the protein by siRNA or CRISPR/Cas9 to demonstrate loss of signal.

### 4.4 Differentiation of H9c2 cells

H9c2 cells are increasingly used as an alternative to cardiomyocytes thus reducing the use of animals. Previous studies have shown that during differentiation of H9c2 cells with retinoic acid, the cardiac l-type voltage-dependent calcium channels is induced as evidenced by expression of the pore-forming α_1C_ subunit as well as cardiac troponin T and troponin I [39], [42]. A recent study performed a transcriptional analysis of the H9c2 cell line in the proliferating and retinoic acid-differentiated state and confirmed altered gene expression including increases in genes associated with mitochondrial energy production [42]. Our results extend this dataset; not only do we demonstrate increases in mitochondrial proteins and a cardiac marker, troponin I as expected, we show a specific increase in the expression of PITPNC1, both at the mRNA and protein level (Figs. 1B and 4D). Adult cardiac myocytes exhibit tissue-specific expression of many proteins and we can now include PITPNC1 as one of them. Our previous studies had identified adult heart as the main tissue that expressed PITPNC1 [30] thus it was initially surprising that H9c2 cells showed little expression of PITPNC1. H9c2 cells proliferate in the presence of serum (10%) but if serum is withdrawn the cells transdifferentiate to a skeletal phenotype. However, if serum withdrawal is accompanied by daily treatment with all-*trans*-retinoic acid, a cardiac phenotype is observed. Expression of PITPNC1 is substantially increased when the cells differentiate towards a mature cardiac phenotype.

Differentiated H9c2 cells remodel their metabolism and instead of relying on glycolysis, the cells become more dependent on oxidative metabolism [41]. This change in energy metabolism requires increased expression of mitochondrial proteins and in H9c2 cells treatment with retinoic acid was found to increase the expression of several mitochondrial markers including GRP75, COXIV and cytochrome C (Fig. 1). The transcriptional co-activator, PGC-1α acts as a master regulator of energy metabolism and mitochondrial biogenesis and differentiation of H9c2 cells with retinoic acid causes a substantial increase in PGC-1α expression [40], [42]. Our results confirm that retinoic acid treatment causes the H9c2 cells towards a cardiac phenotype with differentiation-associated changes in mitochondrial biogenesis [40] and show that PITPNC1 is a new and novel biochemical marker for the differentiated state. Moreover, PITPNC1 was also found highly enriched in the brain, but absent in liver. This suggests that PITPNC1 has tissue-specific functions rather than a house-keeping function. Its investigation in cultured H9c2 cells is hampered because knockdown of proteins during differentiation is technically a problem. Recent work has identified that PITPNC1 is over-expressed in multiple cancer types and facilitates Golgi extension and vesicular secretion of pro-tumour factors in these cancer cells. PITPNC1 was found to associate with RAB1B and aiding the recruitment of GOLPH3 to the Golgi [52].

The majority of studies utilising H9c2 cells as a model for cardiomyocytes do not differentiate the cells [53], [54]. We would suggest that this could distort the results as it is noted that H9c2 cells are increasingly used to study the effects, mechanisms, and therapeutic interventions for hypoxia/re‑oxygenation, autophagy, hypertrophy, insulin resistance, apoptosis, endoplasmic reticulum–mitochondria interactions and cellular signalling studies [54].

## Conflict of interest

The authors declare that they have no conflicts of interest with the contents of this article.

## Transparency document

Transparency document Image 1
